# Effect of Ultra-Small Platinum Single-Atom Additives on Photocatalytic Activity of the CuO_x_-Dark TiO_2_ System in HER

**DOI:** 10.3390/nano15171378

**Published:** 2025-09-06

**Authors:** Elena D. Fakhrutdinova, Olesia A. Gorbina, Olga V. Vodyankina, Sergei A. Kulinich, Valery A. Svetlichnyi

**Affiliations:** 1 Laboratory of Advanced Materials and Technology, Tomsk State University, 634050 Tomsk, Russia; 2 Department of Physical and Colloid Chemistry, Faculty of Chemistry, Tomsk State University, 634050 Tomsk, Russia; 3 Research Institute of Science and Technology, Tokai University, Hiratsuka 259-1292, Kanagawa, Japan

**Keywords:** dark TiO_2_, defects, copper oxide clusters, platinum single-atoms, ultra-small doping, strong metal-support interaction, photocatalysis, hydrogen evolution reaction, pulsed laser ablation

## Abstract

Improving the efficiency of photocatalysts for hydrogen production while minimizing the amount of noble metals used is a pressing issue in modern green energy. This study examines the effect of ultra-small Pt additives on increasing the efficiency of the CuO_x_-dark TiO_2_ photocatalyst used in the hydrogen evolution reaction (HER). Initially, Pt was photoreduced from the hydroxonitrate complex (Me_4_N)_2_[Pt_2_(OH)_2_(NO_3_)_8_] onto the surface of nanodispersed CuO_x_ powder obtained by pulsed laser ablation. Then, the obtained Pt-CuO_x_ particles were dispersed on the surface of highly defective dark TiO_2_, so that the mass content of Pt in the samples varied in the range from 1.25 × 10^−5^ to 10^−4^. The prepared samples were examined using HRTEM, XRD, XPS, and UV-Vis DRS methods. It has been established that in the Pt-CuO_x_ particles, platinum is mainly present in the form of single atoms (SAs), both as Pt^2+^ (predominantly) and Pt^4+^ species, which should facilitate electron transfer and contribute to the manifestation of the strong metal–support interaction (SMSI) effect between SA Pt^n+^ and CuO_x_. In turn, in the Pt-CuO_x_-dark TiO_2_ samples, surface defects (O_v_) and surface OH groups on dark TiO_2_ particles act as “anchors”, promoting the spontaneous dispersion of CuO_x_ in the form of sub-nanometer clusters with the reduction of Cu^2+^ to Cu^1+^ when localized near such O_v_ defects. During photocatalytic HER in aqueous glycerol solutions, irradiation was found to initiate a large number of catalytically active Pt^0^-CuO_x_-O_v_-dark TiO_2_ centers, where the SMSI effect causes electron transfer from titania to SA Pt, thus promoting better separation of photogenerated charges. As a result, ultra-small additives of Pt led to up to a 1.34-fold increase in the amount of released hydrogen, while the maximum apparent quantum yield (AQY) reached 65%.

## 1. Introduction

Photocatalytic hydrogen production, as an alternative energy source, is currently attracting the attention of a wide range of researchers due to its environmental friendliness, the possibility of using solar radiation, and humanity’s growing need for various energy sources [1,2,3]. Photocatalysis is most productive in generating hydrogen not through direct photodecomposition of water, but through the use of aqueous solutions of sacrificial reagents, which are often inexpensive products of renewable biomass processing, such as glycerin [4]. The efficiency of H_2_ evolution in the photocatalytic hydrogen evolution reaction (HER) is largely determined by the properties of the catalysts used. In recent years, many photocatalytic systems based on semiconductors, as well as heterostructures based on them [5,6,7,8], carbon materials [9,10,11,12], metal–organic coordination polymers [13,14], and other materials, have been proposed. Titanium dioxide-based photocatalysts remain one of the most sought-after materials. In addition to its high efficiency and unique physical, chemical, and optical properties, TiO_2_ is non-toxic, chemically stable, and inexpensive [6,15,16].

Doping, increasing defect density, and creating composite catalysts with TiO_2_-based heterojunctions increases the efficiency of the photocatalyst and expands its spectral range [16,17,18]. Materials containing noble metals (Pt, Pd, Ru, and Au) as co-catalysts are known to demonstrate higher activity in the HER process among TiO_2_-based photocatalysts [19,20]. One of the most effective approaches is to increase the dispersibility of the co-catalyst metal down to a single atom (SA) in order to create a large number of active centers and increase quantum efficiency [21,22,23]. High dispersion is also important for minimizing the amount of expensive noble metals used. Surface defects (such as oxygen vacancies and Ti^3+^ ions) play an important role in the dispersion of metal on the titania surface, capturing noble metal particles, which leads to their fixation on the carrier and an increase in dispersion [24,25]. Surface defects also contribute to the strong metal–support interaction (SMSI) effect, which facilitates the transfer of electrons from the semiconductor support to the metal [26,27].

Apart from expensive noble metals, the use of transition metals in a highly dispersed state is currently actively investigated for the modification of TiO_2_ [28,29,30]. In this light, Cu-modified materials show very promising results in photocatalytic hydrogen production [31,32,33,34], while their activity usually still remains lower than when using Pt. To minimize the use of noble metals, among other things, joint modification of TiO_2_ with Cu and Pt was proposed [35,36,37,38,39,40]. In this case, the increase in photocatalytic activity may be associated with both additive [39] and synergistic effects [36,37,38]. Moreover, synergism will vary depending on the method of metal introduction. For instance, Dozzi and coworkers demonstrated high efficiencies of H_2_ production from mixtures of methanol and water vapor achieved through the introduction of Cu(II) into a Pt/TiO_2_ photocatalyst, with the synergistic effect being associated with the formation of Cu^0^ under the action of light and the facilitation of electron transfer to adsorbed protons [36]. Wang et al. developed a PtCu-TiO_2_ sandwich photocatalyst with the introduction of Cu SAs, which showed a quantum efficiency of 99% in the methanol reforming process, where the synergistic effect is associated with the dual role of Cu atoms: as an electron acceptor to promote the transfer of photoelectrons to Pt and as a hole acceptor for the selective oxidation of methanol to formaldehyde with the generation of H_2_ [37]. In ref. [38], the high activity of the Cu–Pt/TiO_2_ alloy photocatalyst was shown to be due to the increased electron density in the Pt component caused by the addition of Cu. Thus, various methods of introduction, dimensional characteristics of the introduced metal co-catalysts, their ratio, and their state on the TiO_2_ surface were shown to play a key role in the process of photocatalytic hydrogen production.

Previously, using the pulsed laser ablation method, we reported on highly defective dark TiO_2_ nanoparticles (NPs) which exhibited high activity in various photocatalytic processes [41]. When further modified with platinum, such dark TiO_2_ NPs also demonstrated high photocatalytic HER efficiency [42,43,44]. For example, we achieved high Pt dispersion (SA and subnanometer clusters) during photoreduction due to the SMSI effect, which led to increased activity and stability of the photocatalyst in the HER process [44], if compared to doping dark TiO_2_ with Pt nanoparticles [34]. In work [43], by blocking the hydrolysis of Pt precursor during photoreduction, the dispersion of Pt atoms was increased to 53% and a maximum apparent quantum yield (AQY) of 0.77 was achieved. Using various methods of modifying dark TiO_2_ with copper, we demonstrated effective self-dispersion of CuO_x_ on the TiO_2_ surface owing to the SMSI effect and the participation of surface OH groups to the level of subnanometer clusters and SA. As a result, a maximum AQY = 0.55 was achieved [33,34].

The present study aimed at increasing the efficiency of the HER photocatalyst based on dark TiO_2_ modified with CuO_x_, with both nanomaterials prepared by laser ablation in water, by adding ultra-small amounts (0.0025–0.02 wt.%) of Pt atoms. Also, we chose a new strategy in which platinum was applied not directly to the titania carrier, but to CuO_x_ nanoparticles that were then dispersed on the TiO_2_ surface. To achieve SA distribution of Pt atoms, we used the photoreduction method and the complex (Me_4_N)_2_[Pt_2_(OH)_2_(NO_3_)_8_] as a precursor. As a result of this approach, a maximum increase in hydrogen yield of 1.34 times was achieved with the addition of only 0.0025 wt.% of platinum.

## 2. Experimental Part

### 2.1. Sample Preparation

#### 2.1.1. Preparation of Dark TiO_2_, CuO_x_, and Pt/CuO_x_ Nanopowders

The starting nanopowders of highly defective dark TiO_2_ and CuO_x_ were obtained by pulsed laser ablation of Ti and Cu metal targets (99.9% purity) placed in a glass cylindrical reactor with distilled water, using focused radiation from an LS-2131M-20 Nd:YAG laser (LOTIS TII, Minsk, Belarus) with parameters λ = 1064 nm, 7 ns, 20 Hz, and 150 mJ. The as-produced colloidal solutions of TiO_x_ and CuO_x_ NPs were dried in open containers in air at a temperature of 60 °C. The TiO_x_ powder was additionally annealed in a muffle furnace for 4 h at a temperature of 400 °C to enhance their crystallinity while maintaining high defectivity. A more detailed description of the synthesis of nanopowders and their physicochemical characteristics is presented in previously published studies [41,42,45,46] for dark TiO_2_ and CuO_x_, respectively.

As a next step, the CuO_x_ nanopowder was then modified with platinum, which was achieved through the photoreduction of the hydroxonitrate complex (Me_4_N)_2_[Pt_2_(OH)_2_(NO_3_)_8_] on its surface [47]. The CuO_x_ powder was placed in an aqueous-alcoholic (ethyl alcohol) solution, to which the platinum precursor, previously dissolved in a minimal amount (100 μL) of acetone, was added. The calculated Pt concentration was 0.5 wt.%. Photoreduction was carried out for three hours under irradiation with a Philips TL 6W BLB fluorescent UV lamp (365 nm) and constant stirring of the solution. The end of the photoreduction process was monitored by the decrease in the intensity of the long-wave absorption band of the complex (Me_4_N)_2_[Pt_2_(OH)_2_(NO_3_)_8_] (see Figure S1). The obtained sample was then washed and dried at 60 °C, being hereafter designated as sample Pt/CuO_x_.

#### 2.1.2. Preparation of XCuO_x_-Dark TiO_2_ and Pt/XCuO_x_-Dark TiO_2_ Catalysts

**Figure 1:**
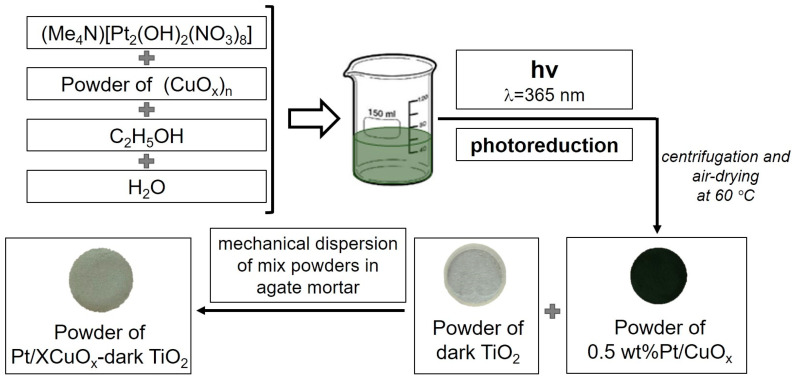
Synthesis scheme for the Pt/XCuO_x_-dark TiO_2_ sample series.

Two series of catalysts, XCuO_x_-dark TiO_2_ and Pt/XCuO_x_-dark TiO_2_ series (where X is the wt.% of CuO_x_), were prepared using the approach previously proposed and described in more detail in ref. [34]. First, dark TiO_2_ powder was mixed in the required proportions with CuO_x_ or Pt/CuO_x_ powders (see Section 2.1.1) and thoroughly homogenized in a planetary mill without balls for 30 min at 300 rpm. The resulting powder mixtures were then subjected to additional mechanical treatment by intensive grinding in an agate mortar for 15 min. As a result, the CuO_x_ content in the XCuO_x_-dark TiO_2_ and Pt/XCuO_x_-dark TiO_2_ samples ranged from 0.25 to 4 wt.%. Accordingly, the platinum content in the samples ranged from 1.25 × 10^−3^ to 2 × 10^−2^ wt.%. The Pt/CuO_x_ ratio remained constant in all samples of the series. The synthesis scheme for the Pt/XCuO_x_-dark TiO_2_ sample series is presented in Figure 1.

### 2.2. Sample Characterization Methods

The crystal structure of the samples was studied by X-ray diffractometry (XRD) using a XRD-7000 diffractometer from Shimadzu (Kyoto, Japan) with a monochromatic CuKα radiation (1.5418 Å) in the 2θ angle range of 10–80° and at a scanning rate of 0.02°/s. The phase composition was analyzed using the PDF-4 database (Release 2022 RDB). To refine the lattice parameters and determine the coherent scattering regions (CSRs) for crystal phases, the POWDER CELL 2.4 full-profile analysis program from Informer Technologies, Inc. (Los Angeles, CA, USA) was used.

The microstructure of samples was studied by transmission electron microscopy (TEM) using a Themis Z double Cs corrected electron microscope from Thermo Fisher Scientific (Waltham, MA, USA) operating at an accelerating voltage of 200 kV. The spectrum imaging results were obtained using a Super-X G2 EDX detector (Thermo Fisher Scientific, Waltham, MA, USA) and a HAADF detector for image registration in scanning (STEM) mode. Crystal lattice values in the obtained (S)TEM images were analyzed using the Fourier method by means of the DigitalMicrograph 3.3.1 Software (Gatan, Inc., Pleasanton, CA, USA). For TEM studies, the samples were dispersed by ultrasound in ethanol and deposited on standard copper grids covered with a holey carbon film.

The elemental composition, chemical and electronic state of atoms on the sample surface were studied by means of X-ray photoelectron spectroscopy (XPS), for which an ES-300 Kratos Analytical tool from Shimadzu (Kyoto, Japan) with non-monochromatic MgKα X-ray radiation (*hν* = 1253.6 eV) was used. To determine the elemental composition and charge states of the elements, survey spectra were recorded at 1 eV intervals, and the spectral regions of the main lines of the elements in study were examined in detail at 0.1 eV intervals. Internal calibration of the spectra was performed using the Ti 2p_3/2_ peak, for which the value of binding energy was taken as 458.6 eV. To determine the charge states from the XPS data, a deconvolution technique was used with individual Gaussian–Lorentzian components or their doublets.

UV-Vis spectra of powders were studied by diffuse reflection in the range of 230–800 nm using a Cary 100SCAN spectrophotometer from Varian (Melbourne, Australia) with a DRA-CA-30I accessory from Labsphere (North Sutton, NH, USA). MgO was used as the standard reflection sample. The reflection spectra were converted using the Kubelka–Munk transformation, and the optical bandgap width (*E*_g_) was determined using the Tauc method with Formula (1):

(*αhν*)^1/*n*^ = *B* (*hν* − *E*_g_) (1)

where *h* is Planck’s constant, *v* is frequency, *B* is the proportionality coefficient, and *n* is a factor depending on the nature of the electronic transition in the semiconductor (being *n* = 2 for indirect bandgap TiO_2_).

### 2.3. Photocatalytic Activity Studies

The photocatalytic properties of the samples were investigated in the hydrogen evolution reaction (HER) carried out in an aqueous solution of glycerol (20 wt.%). A cylindrical quartz reactor was filled with 100 mL of solution and 50 mg of catalyst. The experiment was conducted in a closed gas system using Ar carrier gas. Illumination was provided by a light-emitting diode (LED) with a wavelength of 375 nm through the side walls of the reactor. The total LED optical power was measured by a semiconductor detector PD300UV (Ophir, Jerusalem, Israel) and was 172 mW. The amount of hydrogen formed was determined using a Crystal 5000 gas chromatograph with a thermal conductivity detector (Chromatec, Yoshkar-Ola, Russia). The duration of the photocatalytic experiment was 3 h, with more details of the HER experiments and the photocatalytic setup used described elsewhere [33].

The quantum efficiency (AQY) of the hydrogen evolution process was calculated using Formula (2):

(2) $$AQY=N(H_{2})/N(hv)$$

where *N*(H_2_) was the number of evolved H_2_ molecules and *N*(*hv*) was the number of incident photons.

## 3. Results and Discussions

### 3.1. Pt/CuO_x_ Characterization

**Figure 2:**
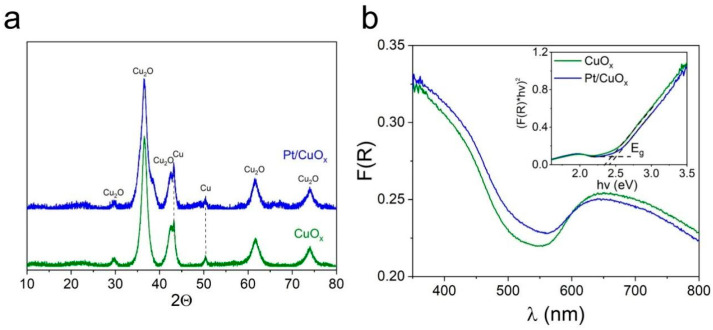
XRD patterns (**a**) and DRS spectra (**b**) of samples CuO_x_ before (green curves) and after Pt photoreduction (blue curves).

Figure 2a compares XRD patterns of samples CuO_x_ and Pt/CuO_x_. The laser-prepared CuO_x_ nanomaterial is characterized by broad reflections corresponding to the cubic crystal lattice of the Cu_2_O phase (PDF4 #01-071-4310), with no reflections corresponding to the CuO phase detected. The size of Cu_2_O crystallites, as calculated using the Debye–Scherrer equation, is ~6–7 nm. The sample also contains a small amount of metallic copper (PDF4 #00-004-0836). After photorecovery, the XRD pattern of the sample remained virtually unchanged (see blue pattern in Figure 2a). No platinum reflections are observed, as its content in the sample is low. According to additional analyses by X-ray fluorescence (XRF), the platinum content in the Pt/CuO_x_ sample was 0.47 ± 0.4 wt.%, which corresponds to the calculated value. In addition, as discussed in more detail below, platinum photo-reduced from the complex (Me_4_N)_2_[Pt_2_(OH)_2_(NO_3_)_8_] was in a highly dispersed state, which is why it could not be detected by XRD.

Figure 2b shows the absorption spectra of CuO_x_ and Pt/CuO_x_ powders. Two absorption bands are observed for the CuO_x_ sample. The absorption in the 400–500 nm range corresponds to the edge of the Cu_2_O absorption band (with a width of *E*_g_ = 2.51 eV, see inset) [48], while the broad absorption band in the 600–800 nm region most likely corresponds to the absorption of CuO (with *E*_g_ ~ 1.4 eV) [49], where the contribution of metallic copper absorption to the long-wave band cannot be ruled out [50]. Photoreduction of platinum on the CuO_x_ surface is seen not to lead to significant changes in the DRS spectrum of the particles, both in terms of the position and intensity of the short-wave and long-wave bands.

**Figure 3:**
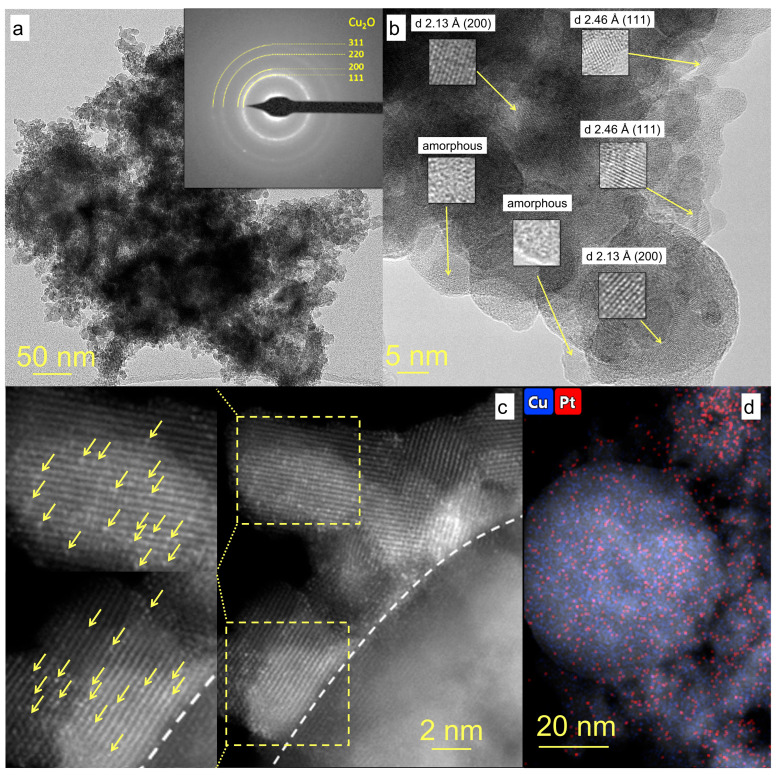
(**a**) TEM images of sample Pt/CuO_x_, with electron micro-diffraction as inset. (**b**) TEM HR with determination of the interplanar distance of the crystal lattice, (**c**) HAADF-STEM with Pt SAs highlighted with yellow arrows. (**d**) EDX mapping image showing distribution of Cu (blue) and Pt (red) elements.

TEM images of Pt/CuO_x_ NPs are presented in Figure 3, where panel (a) shows that the sample consists of agglomerates ranging in size from 100 to 500 nm. The micro-diffraction pattern has the appearance of diffuse rings due to the merging of similar reflections from randomly oriented small crystallites whose positions correspond to the interplanar distances in the Cu_2_O structure (Figure 3a, inset). At higher magnification, HR TEM image in Figure 3b demonstrates that the sample contains both separate small Cu_2_O clusters and larger spherical polycrystalline NPs. The size of Cu_2_O crystallites, both single and in polycrystalline particles, is in the range of 1–5 nm. The image also shows a large number of amorphous CuO_x_ NPs, including those with small crystalline inclusions. Thus, the morphology of copper oxide particles in the Pt/CuO_x_ sample is similar to that of the initial particles without platinum and consists mainly of agglomerates of small (CuO_x_)_n_ clusters [34].

According to high-resolution HAADF-STEM results, Pt is distributed fairly uniformly across the entire surface of the copper oxide NPs (see Figure 3d). However, there are areas of the surface where the Pt concentration is higher. This is most likely due to the more effective fixation of Pt atoms on certain crystal faces of Cu_2_O particles. Platinum is seen to be present in sample Pt/CuO_x_ in an ultra-fine state in the form of single atoms, and no Pt clusters or separate NPs were found. In Figure 3c, for the area above the dotted line, the brightness/contrast was additionally edited and enlarged areas are shown to clearly demonstrate the presence of single Pt atoms (whose position is indicated by yellow arrows).

**Figure 4:**
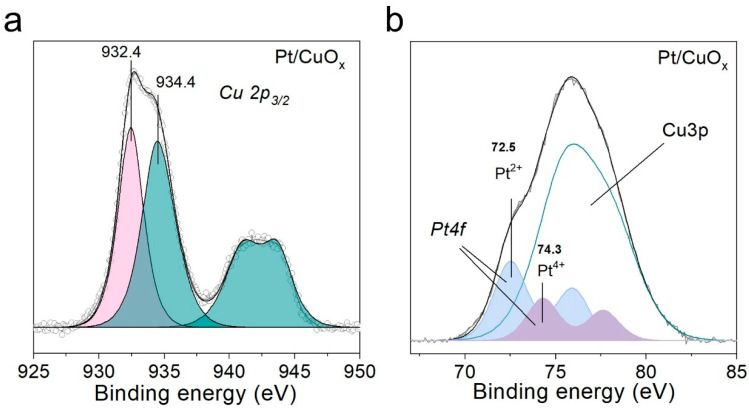
Narrow-scan XPS spectra of Cu 2p (**a**) and Pt 4f (**b**) with deconvolution for sample Pt/CuO_x_.

XPS analysis was conducted to examine the state of chemical elements on the Pt/CuO_x_ surface (Figure 4a,b). Two individual peaks with binding energies around 932.4 and 934.4 eV are observed in the Cu 2p_3/2_ spectra. The former peak corresponds to monovalent copper in the Cu_2_O oxide. Meanwhile, the binding energy value of the latter peak, 934.4 eV, is higher than that characteristic of CuO oxide, which implies that Cu(II) species can also be present in the hydroxide and/or carbonate form. The presence of Cu(II) compounds is also indicated by the clearly expressed satellite structure of the Cu 2p_3/2_ spectrum in the form of shake-up satellite peaks in the binding energy range of 940–945 eV. For a more accurate interpretation of the binding states of copper, Auger spectra were recorded for the Cu *LMM* transition (Figure S2a). The kinetic energy of the Cu *LMM* transition peak maximum was 916.8 eV, which is close to a value characteristic of Cu_2_O oxide. It is worth noting that due to the difference in the kinetic energy of electrons recorded in the case of Cu 2p and Cu *LMM* lines, Cu 2p spectra provide information about more surface states of copper, while Auger spectra correspond to deeper layers. Thus, the XPS data indicate that Cu(II) compounds are present on the surface of Cu_2_O NPs. The application of platinum does not lead to a significant change in the states of copper in the initial sample, whose XPS was investigated earlier in work [34].

The analysis of the chemical states of Pt atoms for sample Pt/CuO_x_ was complicated not only by the low concentration of platinum, but also by the fact that the spectral region of the main Pt 4f peak overlaps with that of copper (Cu 3p region). Nevertheless, this spectral region was decomposed to isolate the contributions of platinum and copper (Figure 4b). The copper contribution was taken into account using the shape of the Cu 3p peak line previously recorded for sample CuO_x_ without platinum. As a result, two chemical states were identified for Pt species, with binding energies in the range of 72.5 and 74.3 eV, which correspond to the oxidized forms of platinum Pt^2+^ and Pt^4+^, respectively. The main state was found to be Pt^2+^, which accounted for ~65% of Pt atoms.

The XPS O 1s spectra for the samples presented in Figure S2b are characterized by a broad peak with a maximum around 531 eV. Oxygen in copper oxides Cu_2_O and CuO is known to have binding energy values of 530.4 and 529.4 eV, respectively, while oxygen in hydroxo- and/or carbonate groups and adsorbed forms of water is characterized by a binding energy value above 530 eV. Thus, analysis of the O 1s spectra indicates the presence of a significant amount of adsorbed oxygen forms on the surface, in addition to oxide oxygen.

Thus, the characterization of Pt/CuO_x_ NPs shows that when platinum is deposited on the surface of CuO_x_ nanopowder by photoreduction from the hydroxonitrate complex (Me_4_N)_2_[Pt_2_(OH)_2_(NO_3_)_8_], Pt atoms are distributed rather uniformly in the form of single atoms in the Pt^2+^ (predominantly) and Pt^4+^ states. At the same time, both the morphology and structure of CuO_x_ particles remain unchanged.

### 3.2. Pt/XCuO_x_-Dark TiO_2_ Characterization

**Figure 5:**
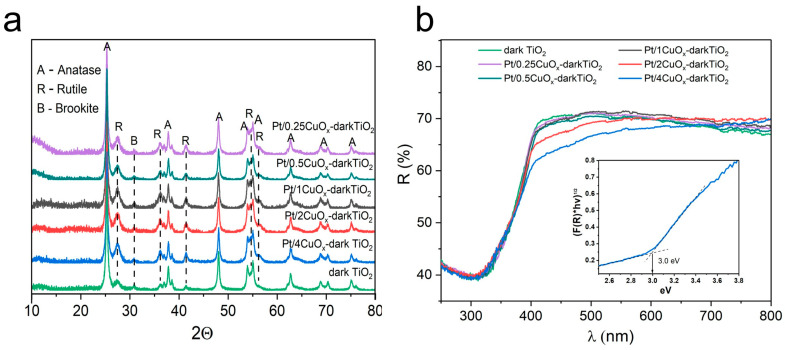
XRD patterns (**a**) and DRS spectra (**b**) for samples Pt/XCuO_x_-TiO_2_.

Figure 5a presents XRD patterns of samples Pt/XCuO_x_-dark TiO_2_, where only reflections corresponding to dark TiO_2_ (consisting mainly of the anatase phase, ~86%, with an average crystallite size of 15–20 nm) are seen. The TiO_2_ particles are also seen to contain rutile and brookite phases, each accounting for ~7%. The specific surface area of the powders was ~86 m^2^/g, and their main type of defects was oxygen vacancies/Ti^3+^ ions [41,43,51].

Modification of the titania surface with Pt/XCuO_x_ NPs was found not to lead to changes in the crystal structure of dark TiO_2_. The width and position of its XRD peaks did not change, which implies that copper was not incorporated into the titania structure [33,34]. As in the case of the XCuO_x_-dark TiO_2_ samples previously studied in work [34], no reflections related to copper compounds were observed in the Pt/XCuO_x_-dark TiO_2_ series, even for the maximum CuO concentration applied in this study (4 wt.%). This is primarily because of the high dispersion of Cu-containing NPs distributed over the surface of TiO_2_ [34] due to the SMSI effect. Additional difficulties in detecting Cu(II) oxide phase reflections are associated with the overlap of the most intense Cu_2_O (111) at 36.5° with that of rutile (101) reflection at 36.3°. At the same time, expectedly, no reflections related to platinum were detected in XRD patterns as its content in the samples did not exceed 0.02 wt.%.

The results of optical studies of the samples using DRS show that all samples of the Pt/XCuO_x_-dark TiO_2_ series have their optical bandgap width *E*_g_ of about 3 eV and weak unstructured absorption throughout the visible spectrum range, which is caused by defects in the TiO_2_ structure (Figure 5b). The intensity of broadband unstructured absorption increases in the long-wave part of the spectrum, which has also been previously noted by others [52,53]. The reason for this could be localized surface plasmon resonance (LSPR) in defective TiO_x_ particles, whose existence in semiconductor materials was discussed in works [54,55]. The presence of CuO_x_ in the form of a shoulder in the spectra begins to appear in the spectra only at concentrations above 1 wt.%, when (CuO_x_)_n_ clusters enlarge on the TiO_2_ surface. This additional absorption in the 400–550 nm range is associated with charge transfer in Cu–O–Cu units in oxide Cu(I) [56].

**Figure 6:**
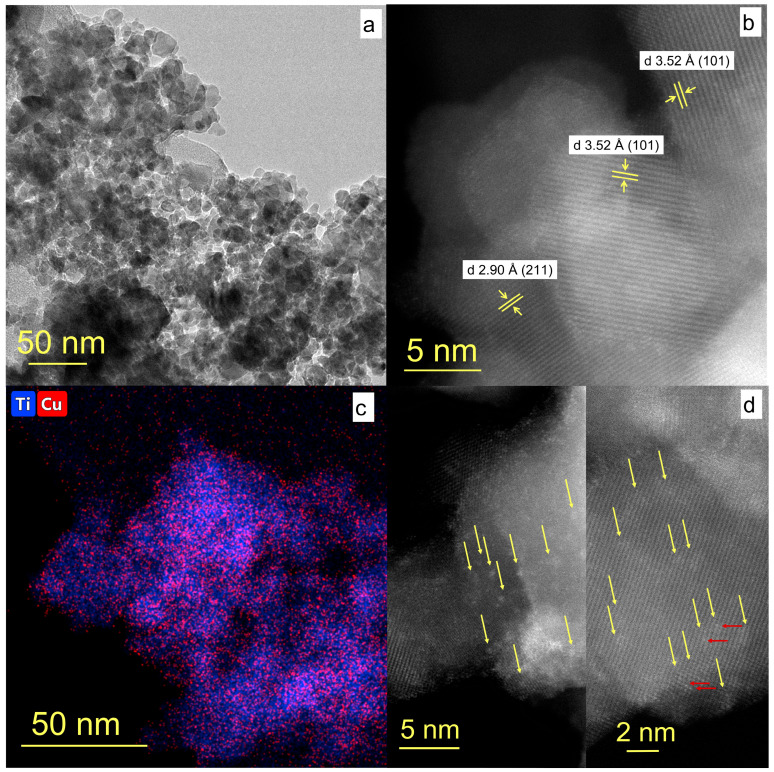
(**a**) TEM image of sample Pt/0.5CuO_x_-TiO_2_. (**b**) HR TEM image of the same sample with interplanar distances of crystal lattice. (**c**) EDX elemental mapping of the sample showing Ti (blue) and Cu (red) local distribution. (**d**) HAADF-STEM images with Pt/0.5CuO_x_ clusters and SA Pt indicated with yellow and red arrows, respectively.

The morphology of composite Pt/XCuO_x_-dark TiO_2_ NPs is shown in Figure 6. The overview low-resolution TEM image presented in Figure 6a shows that the sample consists mainly of agglomerated spherically shaped particles with an average size of ~10–20 nm, which is consistent with the XRD and BET data. The high-resolution image obtained in HAADF-STEM mode shows that the particles have an ordered crystalline structure. Figure 6b shows lattice stripes with *d* = 3.52 Å, corresponding to the crystallographic planes (101) of anatase, the main phase of dark TiO_2_, as well as *d* = 2.90 Å, corresponding to the (211) plane of brookite. No phases related to CuO_x_ NPs were detected by TEM.

EDX elemental mapping presented in Figure 6c shows that copper is fairly evenly distributed across the surface of the titanium dioxide particles. At the same time, the HAADF-STEM image in Figure 6d shows that copper is found on the surface mainly in the form of subnanometer clusters, as in the series of XCuO_x_-dark TiO_2_ samples previously reported elsewhere [34]. Owing to the very low platinum content applied in the series Pt/XCuO_x_-dark TiO_2_, it was not possible to obtain reliable results on its distribution in samples. Copper clusters on the surface of TiO_2_ have relatively low contrast in the HAADF-STEM image, and their location is indicated by yellow arrows in Figure 6d. Meanwhile, brighter/higher-contrast areas in some (CuO_x_)_n_ clusters are associated with the presence of single Pt atoms (indicated by red arrows).

The results of the XPS study of the state of copper and titanium for samples of the Pt/XCuO_x_-dark TiO_2_ series coincide with the results for samples of the XCuO_x_-dark TiO_2_ series previously studied by us (see sample 1CuO_x_-dark TiO_2_-md in work [34]). When dispersed on the TiO_2_ surface, the intensity of XPS Cu peak around 934.4 eV was observed to decrease, which corresponds to Cu(II) compounds. That is, Cu^2+^ ions are concluded to be reduced to Cu^1+^ as a result of the interaction of surface oxygen vacancies/Ti^3+^ ions in dark TiO_2_ with copper clusters to form Cu^1+^-O_v_-Ti^4+^ sites. Expectedly, no ultra-low concentrations of platinum were detected on the TiO_2_ surface by XPS.

Thus, the studies showed that ultra-low concentrations of platinum used in the series samples do not lead to changes in the structure, morphology, and optical properties of the composite NPs when compared to the samples of series XCuO_x_-dark TiO_2_.

### 3.3. Studies of Photocatalyst Activity in HER

Table 1 presents the results of photocatalytic tests conducted on the samples during the hydrogen production reaction from an aqueous glycerol solution. As our previous studies have shown [33,34], when CuO_x_ NPs are applied to dark TiO_2_, the SMSI effect and the participation of surface OH groups result in effective self-dispersion and uniform distribution of small Cu-containing clusters across the titania surface. As a result, CuO_x_-O_v_-darkTiO_2_ positions are formed, at which effective charge transfer and separation occur, leading to increased hydrogen generation. The increase in HER efficiency correlates with an increase in the amount of CuO_x_ introduced up to 1 wt.%. With a further increase in the CuO_x_ content, the HER efficiency and, accordingly, the AQY begin to decrease, which is associated with the enlargement of CuO_x_ clusters and excessive blocking of the TiO_2_ surface.

The results of this study demonstrate that ultra-small platinum additives (within the range of 0.00125–0.005 wt.%) enhance hydrogen yield by more than 1.3 times when compared to Pt-free samples with the same CuO_x_ concentration (see the ratio HER(Cu + Pt)/HER(Cu) in Table 1). It should be noted that both sample CuO_x_ and sample Pt/CuO_x_, which was not deposited onto titania, were not active in HER (see Table 1). The maximum HER productivity of 3.84 mM/g_cat_ and AQY = 65% were achieved for the Pt/1CuO_x_-dark TiO_2_ sample, which is 1.32 times higher than for the corresponding sample without platinum (HER of 2.92 mM/g_cat_ and AQY = 50%). The greatest relative increase in hydrogen evolution efficiency, 1.34 times, was demonstrated by the Pt/0.5CuO_x_-dark TiO_2_ sample with a platinum content of 0.0025 wt.%. H_2_ evolution for this sample over 3 h was 3.51 mM/g_cat_ and AQY = 60%, while for its Pt-free counterpart 0.5CuO_x_-dark TiO_2_, the values were 2.62 mM/g_cat_ and 45%, respectively [34].

Using the photoreduction method from hydroxonitrate complex (Me_4_N)_2_[Pt_2_(OH)_2_(NO_3_)_8_] according to the procedure described in Section 2.1.1, we applied 0.0025 wt.% Pt to the surface of dark TiO_2_, additionally obtaining a sample of 0.0025Pt/dark TiO_2_. A comparison of the total HER efficiency of the 0.50CuO_x_-dark TiO_2_ and 0.0025Pt/dark TiO_2_ samples with the efficiency of the Pt/0.50CuO_x_-dark TiO_2_ sample showed an increase in H_2_ generation when platinum and copper were present in the sample at the same concentrations (see Table 1 and Figure S3). The synergistic effect was achieved due to the SA distribution of Pt on the surface of small CuO_x_ clusters, as follows from the TEM data (see Figure 3 and Figure 6).

An increase in the content of CuO_x_ and platinum in the samples, as in the case of the XCuO_x_-dark TiO_2_ series, leads to the enlargement of CuO_x_ clusters on the TiO_2_ surface and a decrease in photocatalytic activity.

Comparing the photocatalytic properties of the samples obtained in this work in HER with analogs given in the literature [30,57] for catalysts based on Cu-doped titanium dioxide in the form of SA or oxides (TiO_2_-Cu_2_O heterostructure, z-scheme), it can be concluded that the materials obtained in the present work surpass them in efficiency (parameter AQY). For those more interested in the topic, a detailed comparison of the activity of various types of photocatalysts based on Cu-modified TiO_2_ can be found elsewhere [34].

**Figure 7:**
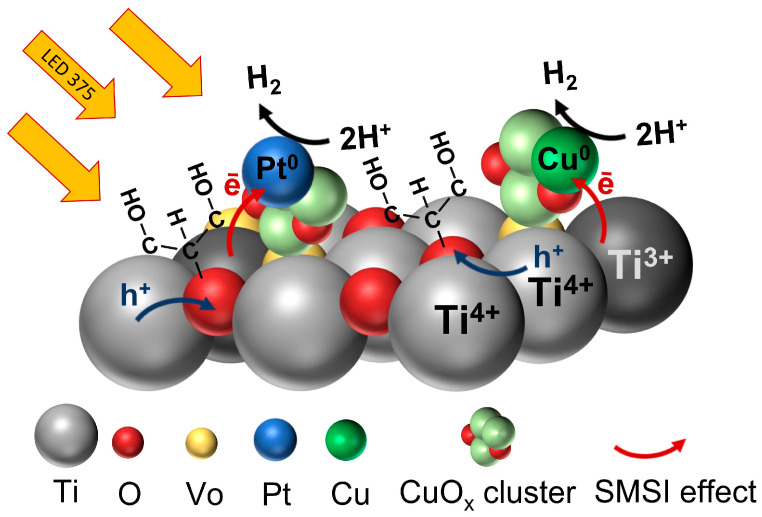
Mechanism of photocatalytic processes on the surface of Pt/XCu_2_O-dark TiO_2_ nanomaterials in HER.

Summarizing the results obtained in this study, it can be stated that the increase in hydrogen generation efficiency with ultra-low platinum additions in the Pt/XCuO_x_-dark TiO_2_ sample series can be associated with the following main factors:

(i). The distribution of low Pt concentrations in the form of SAs on the CuO_x_ surface, achieved during photoreduction, does not interfere with the self-dispersion of copper oxide NPs on the dark TiO_2_ surface and their uniform distribution in the form of subnanometer Pt/CuO_x_ clusters during the subsequent synthesis of Pt/XCuO_x_-dark TiO_2_ photocatalysts.
(ii). A small amount of SA Pt on the CuO_x_ surface does not significantly affect the interaction of CuO_x_ clusters with oxygen vacancies/Ti^3+^ ions in titania and does not hinder the reduction of surface Cu(II) to Cu(I). Therefore, the SA Pt also does not hinder the efficient transfer of electrons between CuO_x_ and dark TiO_2_ in Pt/XCuO_x_-dark TiO_2_ samples.
(iii). Since, in this work, platinum was pre-deposited onto CuO_x_ particles, in our Pt/XCuO_x_-dark TiO_2_ samples, in addition to short-lived Cu^0^-CuO_x_-O_v_-dark TiO_2_ active centers, short-lived Pt^0^-CuO_x_-O_v_-dark TiO_2_ active centers were also formed as a result of photoirradiation. In such centers, due to the SMSI effect, efficient electron transfer to Pt or Cu occurs, which leads to better spatial separation of charges. This is confirmed by photocurrent studies, which showed that the number of photo-generated charges increases in the series dark TiO_2_–CuO_x_-dark-TiO_2_–Pt/CuO_x_-dark-TiO_2_ (see Figure S4). The method for determining photocurrent is described in detail in our previously published works [42,58]. It can be assumed that during irradiation, the photogenerated electron e^−^ is transferred from dark TiO_2_ to a CuO_x_ cluster and/or SA Pt with the formation of short-lived states Cu^0^-CuO_x_-O_v_-dark TiO_2_ and Pt^0^-CuO_x_-O_v_-dark TiO_2_. Then the e^−^ is transferred to a proton H^+^ with the formation of a H_2_ molecule. After irradiation termination, electron transfer and formation of short-lived states Cu^0^ and Pt^0^ cease (see Figure 7).

## 4. Conclusions

In this work, the influence of ultra-small Pt additives on the efficiency of the CuO_x_-dark TiO_2_ photocatalyst in HER was studied. The proposed approach to adding platinum consisted of its preliminary photoreduction on the surface of CuO_x_ nanoparticles, followed by mechanical dispersion of polycrystalline copper oxide nanoparticles on the titania surface. Owing to the effects of SMSI, both between Pt^n+^ and CuO_x_ and between the nanoparticles of CuO_x_ and dark TiO_2_, and the participation of OH groups on the surface, effective self-dispersion of CuO_x_ particles into small clusters CuO_x_ occurred, on which single atoms of Pt were fixed. As a result, during irradiation in photocatalytic HER, in addition to the catalytically active centers Cu^0^-CuO_x_-O_v_-dark TiO_2_, active centers Pt^0^-CuO_x_-O_v_-dark TiO_2_ were also formed, which led to a synergistic effect and an increase in hydrogen evolution efficiency of the catalyst.

The obtained Pt/XCuO_x_-dark TiO_2_ nanomaterials with a platinum content of only 0.00125 to 0.01 wt.% demonstrated a 1.34-fold increase in hydrogen yield, when compared to samples of similar composition without platinum, and the maximum apparent quantum yield reaching 65%. The approaches used in this work to modify TiO_2_ nanoparticles with copper and platinum proved to be promising for the synthesis of effective nanomaterials for photocatalytic HER.

## Figures and Tables

**Table 1:** Photocatalytic characteristics of the studied samples and their performance in HER.

Sample	Cu (wt.%)	Pt * (wt.%)	HER (mM/g_cat_)	AQY	HER (Cu + Pt)/ HER (Cu)/
dark TiO_2_	–	–	0.12	0.02	–
CuO_x_	–	–	0	0	–
Pt/CuO_x_	–	0.5	0	0	0
0.25CuO_x_-dark TiO_2_	0.25	–	2.23	0.38	–
0.50CuO_x_-dark TiO_2_	0.50	–	2.62	0.45	–
1.00CuO_x_-dark TiO_2_	1.00	–	2.92	0.50	–
2.00CuO_x_-dark TiO_2_	2.00	–	2.40	0.41	–
4.00CuO_x_-dark TiO_2_	4.00	–	1.85	0.31	–
Pt/0.25CuO_x_-dark TiO_2_	0.25	0.00125	2.94	0.50	1.32
Pt/0.50CuO_x_-dark TiO_2_	0.50	0.0025	3.51	0.60	1.34
Pt/1.00CuO_x_-dark TiO_2_	1.00	0.005	3.84	0.65	1.32
Pt/2.00CuO_x_-dark TiO_2_	2.00	0.01	2.88	0.54	1.20
Pt/4.00CuO_x_-dark TiO_2_	4.00	0.02	1.87	0.31	1.01
0.0025Pt/dark TiO_2_	–	0.0025	0.66	0.11	–

* Calculated amount of injected Pt.

## Data Availability

The data presented in this study are available upon request from the corresponding authors.

## Supplementary Materials

The following supporting information can be downloaded at https://www.mdpi.com/article/10.3390/nano15171378/s1, Figure S1: Absorption spectra of complex (Me4N)_2_[Pt_2_(OH)_2_(NO_3_)_8_] decomposition before and after irradiation; Figure S2: Auger spectra of Cu *LMM* (**a**), XPS of oxygen O 1s with deconvolution (**b**); Figure S3: HER for Pt/0.5CuO_x_-dark TiO_2_ in comparison with reference samples; Figure S4: Transient photocurrent responses for samples in Na_2_SO_4_ electrolyte with glycerol addition.
