# Supplementary material for: Effect of Ultra-Small Platinum Single-Atom Additives on Photocatalytic Activity of the CuOx-Dark TiO2 System in HER
Source: Nanomaterials (Basel). 2025 Sep 6;15(17):1378. doi: 10.3390/nano15171378 (PMC12429992; doi:10.3390/nano15171378)
Supplement: Supplementary file 1 [file nanomaterials-15-01378-s001.zip › nanomaterials-3840126-supplementary.pdf]

Support information for:

Effect of Ultra-Small Platinum Single-Atom Additives on Photocatalytic Activity of the CuO_x-Dark TiO_2 System in HER

Elena D. Fakhrutdinova [1,2], Olesia A. Gorbina[1], Olga V. Vodyankina[2],
Sergei A. Kulinich [3,*], Valery A. Svetlichnyi [1,2,*]

[1] Laboratory of Advanced Materials and Technology, Tomsk State University, Tomsk 634050, Russia
[2] Department of Physical and Colloid Chemistry, Faculty of Chemistry, Tomsk State University, Tomsk 634050, Russia
[3] Research Institute of Science and Technology, Tokai University, Hiratsuka, Kanagawa 259-1292, Japan

* Correspondence: skulinich@tokai.ac.jp (S.A.K.), v_svetlichnyi@bk.ru (V.A.S.)

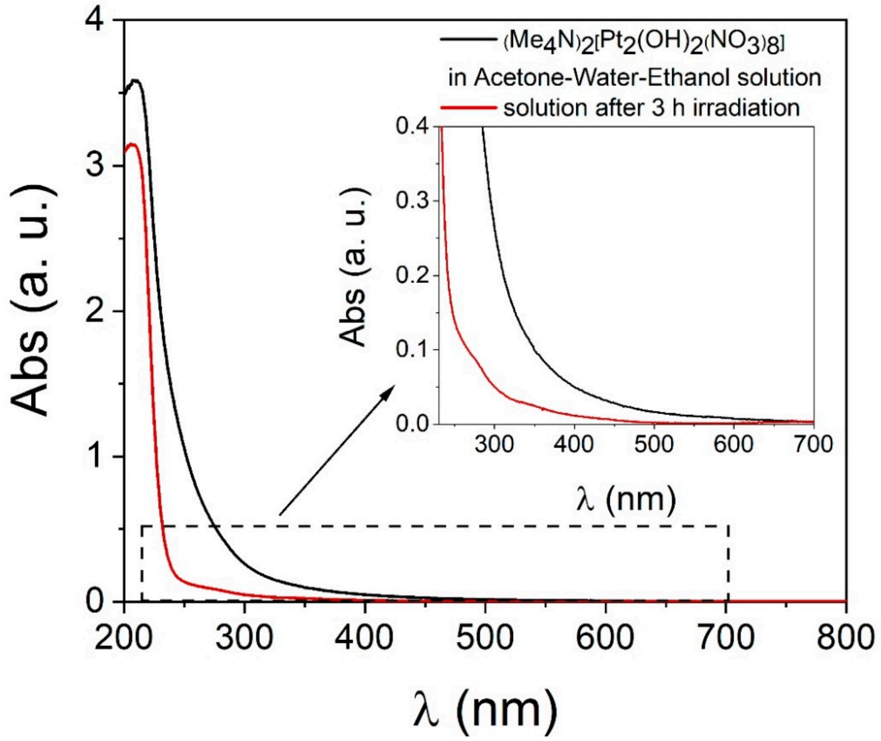

Figure S1. Absorption spectra of complex $(Me_4N)_2[Pt_2(OH)_2(NO_3)_8]$ recorded before and after irradiation.

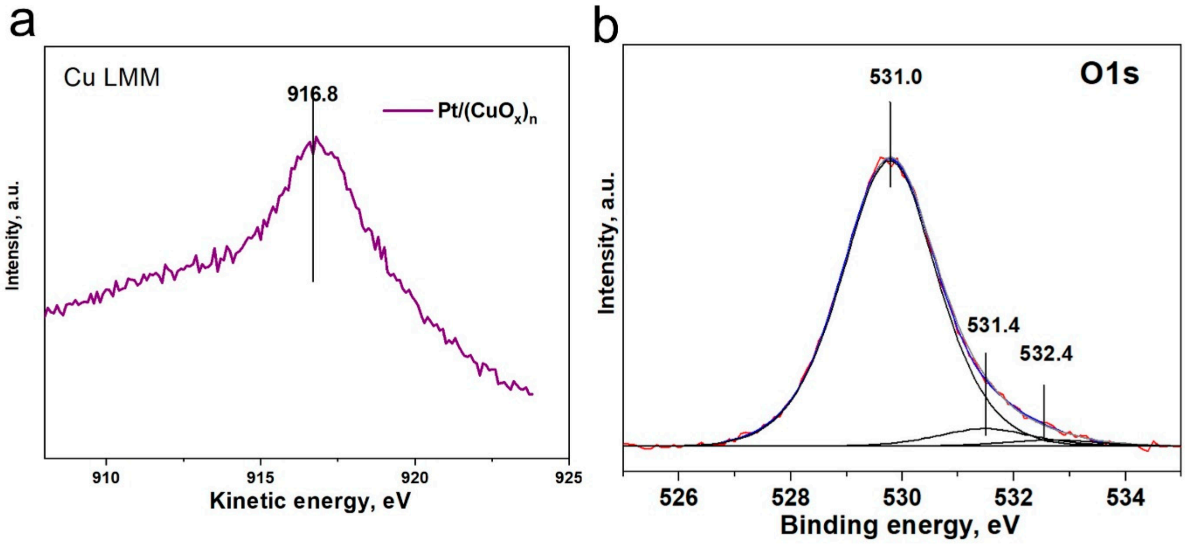

Figure S2. Auger Cu *LMM* spectra (a), XPS O 1s spectrum with its deconvolution (b).

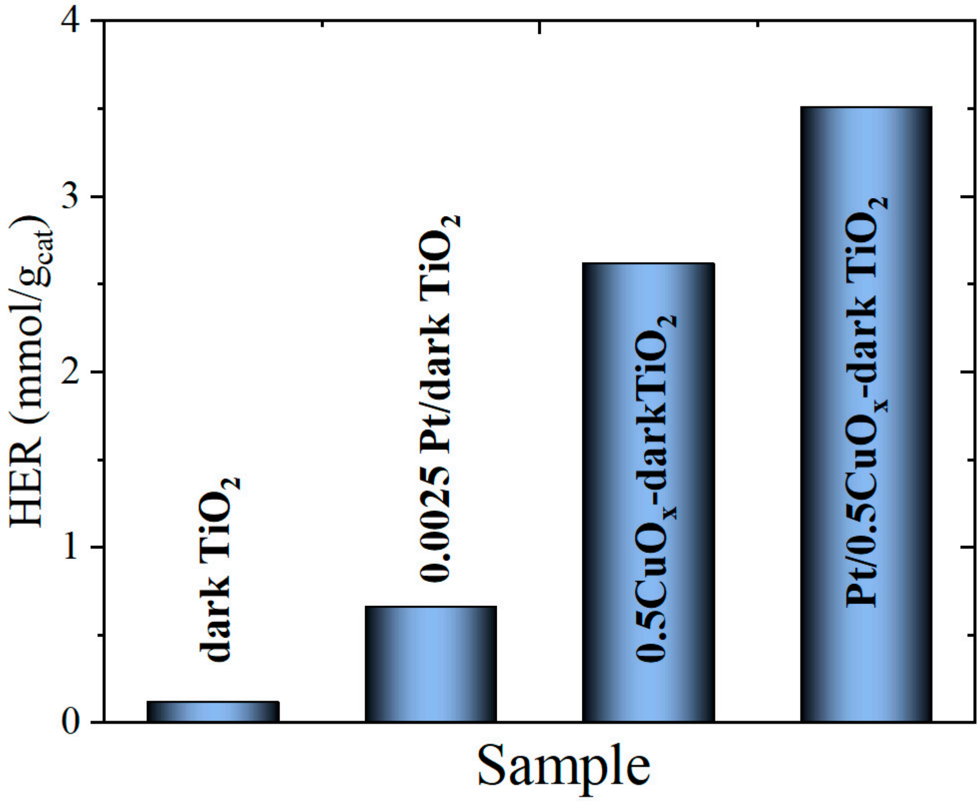

Figure S3. HER for Pt/0.5CuO$_x$-dark TiO_2 in comparison with reference samples.

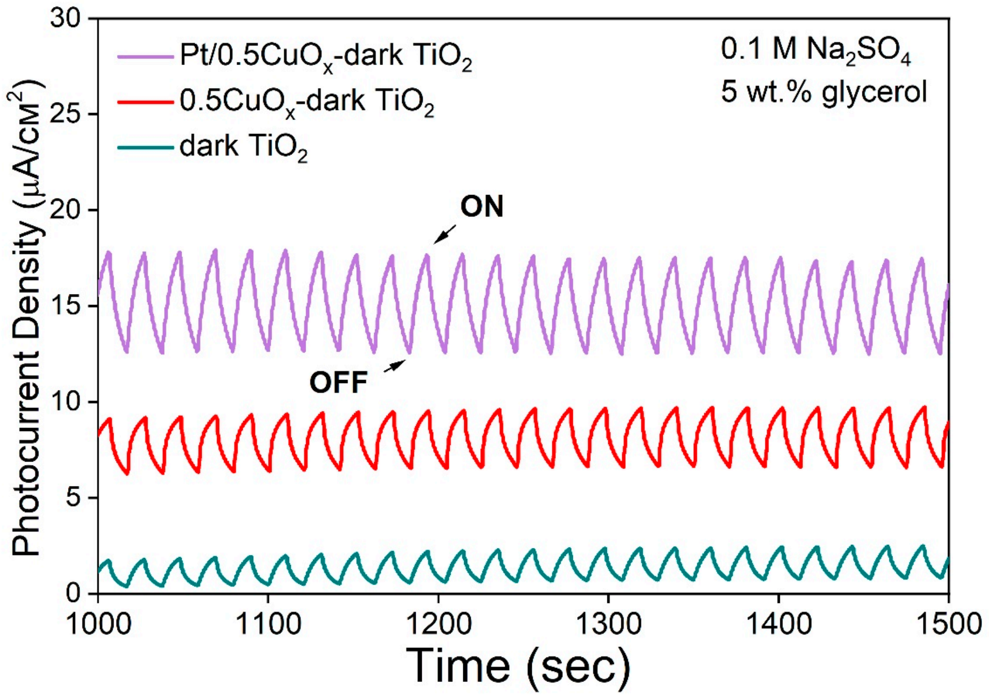

Figure S4. Transient photocurrent responses for samples in Na_2SO_4 electrolyte with glycerol addition.
